# A Retrospective Observational Study to Assess Prescription Pattern in Patients with Type B Aortic Dissection and Treatment Outcome

**DOI:** 10.1155/2016/5173898

**Published:** 2016-08-01

**Authors:** Kuang-Ming Liao, Chung-Yu Chen, Shih-Han Wang, Jiann-Woei Huang, Chen-Chun Kuo, Yaw-Bin Huang

**Affiliations:** ^1^Department of Internal Medicine, Chi Mei Medical Center, Chiali, No. 606, Jialixing, Jiali District, Tainan 72263, Taiwan; ^2^Department of Pharmacy, Kaohsiung Medical University Hospital, No. 100, Tzyou 1st Road, Sanmin District, Kaohsiung 80708, Taiwan; ^3^School of Pharmacy, Master Program in Clinical Pharmacy, Kaohsiung Medical University, No. 100, Shih-Chuan 1st Road, Sanmin District, Kaohsiung 80708, Taiwan; ^4^Division of Cardiovascular Surgery, Department of Surgery, Kaohsiung Medical University Hospital, No. 100, Tzyou 1st Road, Sanmin District, Kaohsiung 80708, Taiwan

## Abstract

Aortic dissection is a life-threatening condition. However, the use of medication to treat it remains unclear in our population, particularly in patients with a type B aortic dissection (TBAD) who do not receive surgery. This retrospective cohort study evaluated antihypertensive prescription patterns and outcomes in patients with nonsurgical TBAD. We reviewed the hospital records of patients with TBAD at a medical center in Taiwan from January 2008 to June 2013 to assess the baseline information, prescribing pattern, event rate, and clinical effectiveness of different antihypertensive treatment strategies. A Cox proportional hazards model was used to estimate outcomes in different antihypertensive strategies. The primary endpoints were all-cause mortality and hospital admission for an aortic dissection. We included 106 patients with a mean follow-up period of 2.75 years. The most common comorbidity was hypertension followed by dyslipidemia and diabetes mellitus. Study endpoints mostly occurred within 6 months after the index date. Over 80% of patients received dual or triple antihypertensive strategies. Patients treated with different treatment strategies did not have a significantly increased risk of a primary outcome compared with those treated with a monotherapy. We found no significant difference in the primary outcome following the use of different antihypertensive medication regimes.

## 1. Introduction

An aortic dissection is a life-threatening condition that is associated with high rates of morbidity and mortality in both the developed and developing countries worldwide [1]. An aortic dissection is classified according to its anatomical location and time from onset. A Stanford classification type A aortic dissection involves the ascending aorta and requires surgery. Type B aortic dissections (TBAD) originate in the descending aorta, regardless of any retrograde involvement of the aortic arch and do not involve the ascending aorta [2]. TBAD can be clinically managed under most conditions, often without requiring surgery. The acute phase is defined as the 14-day period following the initial onset of symptoms because of high morbidity and mortality rates. The chronic phase is defined as symptoms lasting for >2 weeks during which the patient's condition has remained stable [1]. A study revealed TBAD mortality rates to be 10.7% in patients who were treated with medication and 31.4% in patients who underwent surgery [1]. Medication is essential for controlling TBAD, the goals of which are to lower the systolic blood pressure (BP) and heart rate, slow the progression of the disease, and reduce the associated morbidity and mortality.

There are two treatment guidelines for the control of high BP in aortic dissection. The European Society of Cardiology guidelines [3] recommend beta-blockers and also suggest treatment with calcium antagonists, although there are no available supporting data for this indication. Vasodilators are another option for high BP, although their use should be combined with beta-blockers to avoid reflex tachycardia. The other available treatment guidelines were from Japan in 2006, which were updated in 2011, although they lack robust evidence regarding their effectiveness in aortic dissection [4]. The International Registry of Acute Aortic Dissection (IRAD) database revealed that beta-blockers and calcium channel blockers were associated with improved survival in patients with aortic dissection. However, this benefit was not observed for angiotensin-converting enzyme inhibitor (ACEI) therapy [5]. The IRAD analysis speculated that different types of antihypertensive agents could have a range of different therapeutic efficacies. This study aimed to assess antihypertensive treatment effects in patients with TBAD by determining antihypertensive prescription patterns and TBAD outcomes without surgery. The study was based in a medical and research center hospital in Taiwan.

## 2. Methods

### 2.1. Patient Population

We retrospectively reviewed the medical records of patients with TBAD at our institute between January 2008 and June 2013. The study protocol was approved by the Institutional Review Board of the Kaohsiung Medical University Hospital (October 4, 2013; KMUH-IRB-20130199). This retrospective cohort study did not require patient informed consent, in accordance with the current rules of our hospital.

Nonsurgical TBAD cases were reviewed by an experienced investigator who was not allowed to participate in the collection of clinical outcome and procedure data. An aortic dissection was primarily defined as two outpatient or one discharge diagnosis of aortic dissection (ICD-9 code: 441) and first-time hospitalization for an aortic dissection from the medical records in our follow-up period. We selected patients with an aortic dissection who were aged over 18 years and who were discharged alive following a first-time hospitalization and who had a medical record in our hospital of over 28 days. We defined the first-time hospitalization date as the index date. We enrolled patients who underwent computer tomography (CT) or angiography. Patients with an unconfirmed aortic aneurysm or aortic dissection who received aortic disease surgery or who had undergone imminent elective surgery before the index date or first-time hospitalization were excluded. Cases of an uncomplicated TBAD were included, whereas patients with a type A aortic dissection, an aortic aneurysm, Marfan syndrome, or a secondary cause of aortic dissection (including trauma, infection, and aortitis) were excluded from our study population. Patients whose smoking status was not recorded or who had incomplete medical records were also excluded from our study population.

### 2.2. Baseline Information and Comorbidity

One well-trained researcher and one cardiologist or cardiovascular surgeon initially measured the largest short axial diameter of the aorta (expect for the aortic arch) from first-time hospitalization CT images and recorded this as the maximum aortic diameter. If the maximum aortic diameter occurred at the aortic arch, the diameter perpendicular to the aortic arch curvature was recorded as the maximum aortic diameter. Maximum aortic diameters were subgrouped into <30 mm, 30–39 mm, 40–49 mm, and ≥50 mm categories. Furthermore, the patients' body mass index (BMI) was defined as one of four categories: underweight (<18.5), standard (≥18.5 to <24), overweight (≥24 to <27), and obese (≥27). We evaluated the smoking status at the index date and current smokers were defined as patients who had smoked 100 cigarettes in their lifetime and currently smoked cigarettes every day (daily) or on some days (nondaily).

BP and heart rate were followed from first-time hospitalization and during the follow-up period. The evaluated BP in all patients included systolic blood pressure (SBP), diastolic blood pressure (DBP), and mean atrial pressure (MAP). The baseline BP and heart rate were measured two days before discharge from the first-time hospitalization. Patients returned for outpatient visits every three to 6 months depending on the stability of their clinical condition. Follow-up BP and heart rate data at regular follow-up appointments (as outpatients) with a cardiologist or cardiovascular surgeon after discharge were averaged over a year. In our study, we defined poor BP control as an average SBP ≥ 140 mmHg or average DBP ≥ 90 mmHg, and poor heart rate control was defined as a heart rate ≥ 80 beats/min [6].

Comorbidities were extracted from our medical data within a year before the index date by the ICD-9 code system. Comorbidity was defined as having at least one hospitalization diagnosis or two ambulatory diagnoses. Diseases included hyperlipidemia, hypertension, diabetes mellitus, chronic obstructive pulmonary disease, heart failure, coronary artery disease, cerebrovascular disease, chronic kidney disease, peripheral vascular disease, and any cancer.

### 2.3. Drug Use and Prescribing Pattern

The medications that had been used for aortic dissection in our retrospective study had the following anatomical therapeutic chemical classifications: angiotensin II receptor blocker (ARB) (C09CAxx), ACEI (C09AAxx), calcium channel blocker (CCB) (C08CAxx, C08DB01, and C08DA01), beta-blockers (C07ABxx, C07AAxx, and C07AGxx), diuretics (C03CA01, C03AAxx, C03BAxx, and C03DAxx), *α*-blocker (C02CA04, G04CA03, and G04CA02), vasodilator (C01DX16, C01DAxx, and C02DB02), direct renin inhibitor (C09XAxx), centrally acting *α*_2_-agonists (C02ABxx and C02ACxx), antiplatelet agents, including aspirin, clopidogrel, and dipyridamole (B01AC06, B01AC04, B01AC07, and B01AC16), lipid-lowering agents [HMG-CoA reductase inhibitors (statins)] (C10AAxx) and other lipid-lowering drugs (C10ACxx, C10ABxx, C10ADxx, and C10AX09), oral diabetes drugs (A10BBxx, A10BXxx, A10BGxx, A10BHxx, A10BAxx, and A10BFxx), and anticoagulants (B01AA03, B01AB05, and B01AF01).

For the prescribing pattern, a medication user was defined as a patient receiving treatment for over 1 month or over 28 cumulative daily doses in each period (taken from 1 year before the index date or until one year after the index date). Moreover, patients were categorized as monotherapy, dual therapy, triple therapy, quadruple therapy, and nonusers to evaluate the hazard outcome in our study. To decrease the bias from switching medications in each period and misclassifying our population, we used a medication possession ratio (MPR) of >80% during the follow-up to assist in medical group categorization. For example, if a patient only received beta-blockers with a >80% MPR, the patient was defined as receiving monotherapy in our study. Patients receiving ACEIs and ARBs with >80% MPRs were defined as dual therapy.

### 2.4. Clinical Outcomes

The primary endpoints of this study were composite outcome, including all-cause mortality and admission to hospital because of an aortic dissection. All-cause death was defined as mortality from any cause. We also evaluated secondary endpoints including (i) hospitalization associated with an aortic dissection, (ii) all-cause mortality, and (iii) being referred for surgery repair. These secondary endpoints were estimated separately over time.

### 2.5. Statistical Analysis

Continuous variables are expressed as the mean ± standard deviation and categorical variables as numbers and percentages. Statistical significance was inferred as a two-sided *p* value of <0.05. Each case was followed up until the first occurrence of the predefined outcome or until the end of the follow-up period. Cases in which none of the outcomes had occurred by the end of June 2013 or where death was recorded during the follow-up period were defined as censored.

The incident event rate for each outcome during the follow-up period was calculated by the total number of outcomes that occurred for each outcome (numerator) divided by the total number of cases of each outcome (denominator). The incidence was expressed as the number of cases per 100 person-years. We calculated the event rate in the total population and in patients with different treatment regimens (nonuser, monotherapy, dual therapy, triple therapy, and quadruple therapy). We also divided our follow-up period into ≤6 months, 6 months to 1 year, 1 year to 3 years, and ≥3 years and evaluated the event rate in each follow-up period. We used two-tailed Cochran-Armitage test to evaluate the significance of annual trends in BP and hear rate control from the first year to fourth year.

Univariate and multivariate models were used to estimate hazard ratios (HR) in Cox proportional hazards models to describe the associations of primary outcomes with different treatment strategies in the follow-up period. Models were adjusted for age, gender, baseline maximum aortic diameter, current smoker status, BMI, comorbidities, and poor BP and heart rate control. Analyses and calculations were performed using SAS software ver. 9.3 (SAS Institute, Inc., Cary, NC, USA).

## 3. Results

**Figure 1:**
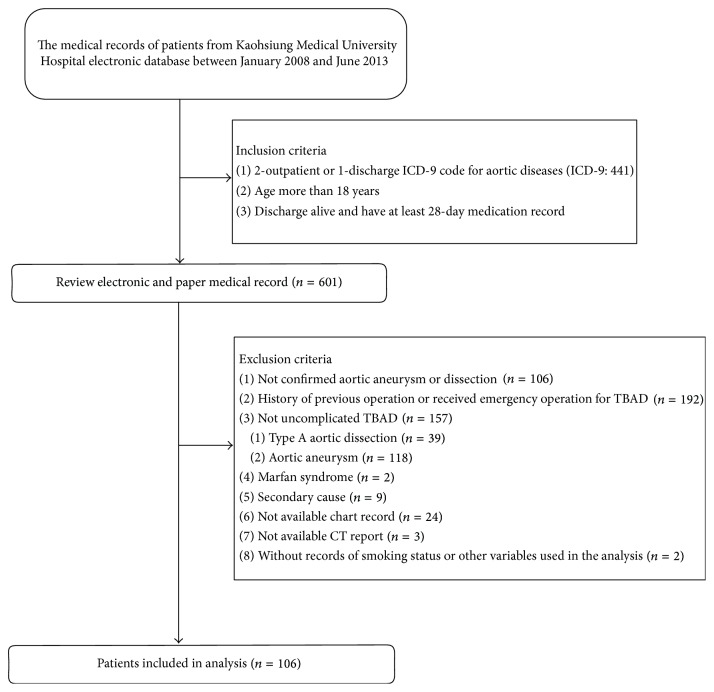
Patient flow diagram of hospital-based cohort study.

Figure 1 showed that the study population was comprised of 106 patients with TBAD. The mean age was 63.4 ± 12.9 years. The study population was 82.1% male (87 patients) and 17.9% female (19 patients). The current smoking rate was 34.9% and the rate of acute aortic dissection was 79.3%. The percentage of underweight, overweight, and obese patients with TBAD was 33%, 32.1%, and 31.1%, respectively. The mean follow-up duration was 2.75 ± 1.64 years. The most common TBAD location was the descending thoracic and abdominal aorta, followed by the thoracic aorta and abdominal aorta. The initial maximum aortic diameter was 4.11 cm in the thoracic aorta and 44.3% of patients had an initial maximum aortic diameter between 3.0 and 3.9 cm. The SBP, DBP, MAP, and heart rate at baseline were 129.2 ± 14.5 mmHg, 76.3 ± 9.7 mmHg, 93.9 ± 10.4 mmHg, and 71.4 ± 9.3 beat/min, respectively. More than 91% of patients with TBAD had a history of hypertension and 18.9% had a history of hyperlipidemia. TBAD occurred predominantly in male patients who were overweight or obese. The nonsurgical characteristics of patients with TBAD are shown in Table 1.  Table 2 shows the number of cases in first year was 106, in second year was 77, in third year was 55, and in fourth year was 33 at study follow-up period. From the first year to fourth year, the poor BP control rate of TBAD cases ranged from 19.8 to 48.5 (*p* value for trend: *p* < 0.001). Otherwise, the poor heart rate control rate ranged from 13.2 to 24.2 (*p* value for trend: *p* < 0.001). The peak poor BP (SBP/DBP) and heart rate control during follow-up period were 151/90 mmHg and 95 beat/min in the fourth year.

Table 3 shows the antihypertensive drug prescribing patterns in patients with nonsurgical TBAD. The antihypertensive treatment was divided into three categories according to the drug class including beta-blockers, ACEIs, ARBs, and CCBs. Beta-blockers were divided into nonselective (propranolol), selective (atenolol and bisoprolol), and combined alpha/beta-blockers (carvedilol and labetalol). CCBs were divided into two classes: the nondihydropyridines (diltiazem and verapamil) and dihydropyridines (remaining CCBs). The most common types of blood pressure prescriptions after the index date were CCBs (88.7%) and beta-blockers (80.2%). From Table 3, only 14% nonsurgical TBAD cases in our population used statin before index date. However, a half of cases were prescribed statin after index date. The most commonly prescribed statin was atorvastatin and rosuvastatin.

Table 4 shows the outcomes of patients with nonsurgical TBAD. In the first 6 months, the event rates were primary endpoint (88.3%), hospitalization associated with an aortic dissection (77.8%), being referred for surgery repair or death (60.0%), all-cause mortality (33.3%), and being referred for surgery repair (40.0%). Compared with the other follow-up time periods, there were significantly higher incident rates for the outcomes of hospitalization associated with aortic dissection, being referred for surgery repair, and all-cause mortality within the first 6 months.

Table 5 shows antihypertensive combinations in patients with nonsurgical TBAD and their event rates over the four year follow-up period. The most commonly prescribed medication regime for lowering BP was treatment with three antihypertensive medications (prevalence: 39.6%), followed by treatment with two antihypertensive drugs (prevalence: 29.3%). Based on the different antihypertensive drug treatment strategies, nonusers had a higher primary outcome event rate (45.5%) compared with the other drug treatment strategies. Otherwise, there was no significant increase in the incidence of overall events from monotherapy to quadruple therapy.

Table 6 shows the univariate and multivariate analyses for all endpoints of each antihypertensive drug treatment strategy. The results revealed that patients with TBAD who were treated with different treatment strategies during the follow-up did not have a significantly increased risk of a primary outcome compared with those treated with monotherapy, after adjusting for the following factors: age, gender, comorbidity, and important risk factors (Table 4). The adjusted HR and primary endpoint were 3.31 for no therapy (95% CI, 0.64–17.22; *p* = 0.156), 1.12 for dual therapy (95% CI, 0.24–5.28; *p* = 0.886), 1.45 for triple therapy (95% CI, 0.33–6.51; *p* = 0.624), and 1.07 for quadruple therapy (95% CI; 0.18–6.39, *p* = 0.944) when compared with monotherapy. As expected, patients who had a larger baseline maximum aortic diameter (aHR = 2.11; *p* < 0.001) had an increased hazard of a primary outcome. However, patients with other risk factors did not have a decreased hazard of primary outcome. Baseline aortic diameter was the only factor that affected patient outcome.

## 4. Discussion

This is the first study to assess prescription patterns in patients with nonsurgical TBAD and to analyze the association between the form of treatment and patient outcome. In our study, the mean age of patients with TBAD was 63.4 years, and the predominance of male cases was similar to previous studies [7, 8]. Most studies do not provide information regarding the BMI of patients with an aortic dissection, although one study revealed that a BMI of ≥25 was significantly related to postoperative hypoxemia in acute type A aortic dissection [9]. In our study, one-third of patients were underweight, one-third were overweight, and one-third were obese. The most common location of an aortic dissection was in the thoracic and abdominal aorta, comprising 77.4% of the study population. The average initial maximum thoracic diameter was 4.11 cm, which was larger than the abdominal aorta diameter. The diameter of the aorta may be influenced by age, gender, body weight, and height [10]. Similar to a previous study, half of the total population had a maximum aortic diameter > 40 mm [11]. The three leading causes of comorbidities were hypertension, hyperlipidemia, and diabetes mellitus.

It has been recommended that patients with TBAD without complications (e.g., without aneurysmal expansion, organ ischemia, and medical treatment failure with refractory pain) are treated with antihypertensive medication during the acute phase, and a surgical treatment is reserved for when the aortic diameter dilatation occurs during the chronic phase. This is because a higher mortality rate is associated with patients receiving surgery during the acute phase [12–19].

The goal of TBAD treatment is to lower a high BP and to control pain. Most studies recommend aiming to maintain the SBP at <120 mmHg and that the first line of medication should be beta-blockers [3]. In our study population, the mean baseline SBP was 129 mmHg. The number of cases having poor BP control steadily increased each follow-up year, from 19.8% in the first year to 48.5% after 4 years observation. Similarly, in the first year the SBP and DBP were 149 mmHg and 86 mmHg, respectively, which increased to 151 mmHg and 90 mmHg in the fourth year. A similar worsening trend was also found regarding heart rate control.

In a previous study [20], 56.6% of people with hypertension receiving treatment were controlled if their average BP was <140/90 mmHg in patients without diabetes. In our study, a poorly controlled BP was a common finding in the outpatient department in patients with TBAD. The level of poor BP control (average BP was >140/90 mmHg) in the first year was 19.8%, which increased during the observation period and was associated with a 2.5-fold increased risk of poor BP control in the fourth year. An awareness of and improved control rates of hypertension are important in patients with TBAD. The importance of effective BP control in these patients should be emphasized because it helps to prevent complications, including arterial occlusion, end-organ damage, and rupture; this latter complication is almost invariably fatal. With this in mind, physicians are required to overcome clinical inertia and aggressively control hypertension to reach the BP target in patents with TBAD.

TBAD medical treatments primarily focus on the management of pain and BP. Beta-blockers and arterial vasodilators if required are used for controlling BP where SBP between 100 and 120 mmHg is the recommended target [19]. In our population, 80.2% of patients had received beta-blockers and 88.7% had received CCBs at some point after their index date. We found that beta-blockers were the most commonly prescribed drugs as a monotherapy, dual therapy, or triple therapy followed by a prescription of CCBs. Therefore, most clinicians appeared to accept the guideline recommendations to use beta-blockers to control BP in patients with TBAD. Forty percent (42/106) of patients received a triple therapy and 11% of patients received a quadruple therapy to control their BP during the study. This phenomenon demonstrates that >50% of patients with TBAD required treatment with three or more antihypertensive medications to achieve the recommended BP control target level. Using univariate and multivariate analyses, we compared the use of different antihypertensive drugs with outcomes, including hospitalization associated with an aortic dissection, being referred for surgery repair or death, and all causes of mortality. We found that there was no statistically significant difference between the outcome and treatment strategy.

A situation of “therapeutic inertia” has been increasingly identified in studies [21]. This indicates that patients and physicians recognize the importance of managing hypertension, dyslipidemia, and diabetes and that effective treatments are available, but physicians do not use a sufficiently intensive therapy. Physician- and patient-related factors contribute to uncontrolled BP, despite the goals for BP management being well defined [21]. Therapeutic inertia was also observed in the critical TBAD population, with the percentage steadily increasing over time. This implies that even a serious condition may be neglected over time. A prompt diagnosis and emergency treatment are critical for aortic dissection, along with the continued monitoring and control of BP after the acute stage. This issue of long-term BP control is worthy of a further study. The prescribing pattern of antihypertensive treatment to patients with TBAD varies globally. Evidence-based guideline updates, education on prescribing behavior for clinicians, and a greater adherence to clinical guidelines may each contribute to reducing aortic dissection complications.

There are many studies that have investigated the relationship between statins, aortic aneurysm, and aortic dissection. Taylor et al. [22] found that preoperative statin use was associated with a lower risk of ascending aortic dilatation in patients with a bicuspid aortic valve. Angeloni et al. [23] retrospectively reviewed 1348 patients in an outpatient clinic from September 2005 to December 2011 and they showed that a statin treatment was associated with a reduced growth rate of ascending aorta aneurysms. Gokani et al. [24] showed that statins may decrease the risk of the devastating consequence of an abdominal aortic aneurysm. A database study demonstrated that statins were significantly associated with improved survival 1 year after abdominal aortic aneurysm repair [25]. In our study, besides antihypertensive medications, statins were one of the common widely prescribed medications after TBAD was diagnosed. After univariate and multivariate analyses for the primary endpoint, only the aortic diameter was associated with the outcome in patients with TBAD. The benefits of statins in patients with an aortic aneurysm or aortic dissection were based on a retrospective study. Further prospective studies are necessary to assess the pleiotropic effects of statins on those with an aortic aneurysm.

Trimarchi et al. [26] used IRAD data to analyze 365 patients with TBAD. Patients with recurrent and/or refractory pain or refractory hypertension (requiring more than two different classes of antihypertensive medications at maximal tolerated doses) had a greater mortality than patients without clinical complications. Their multivariate logistic regression model also demonstrated that recurrent and/or refractory pain or refractory hypertension was a predictor of in-hospital mortality. In our study, there was no statistically significant difference in mortality between antihypertensive monotherapy and antihypertensive quadruple therapy if BP was controlled. No matter which type of medication is chosen, it is always important to reduce BP to within a normal range.

One of the limitations of this study was that it was performed in a single center; however, an adequate sample size was obtained over the 4 years of data collection. The cases reported are definite descriptions and display clear effects after a comprehensive survey and long-term follow-up. In addition, we limited our study to include subjects newly diagnosed with TBAD and who did not have surgery, meaning that the prescription patterns of type A aortic dissection and patients with TBAD receiving surgery should be similarly investigated.

## 5. Conclusions

Our study indicates that, in this Asian population, the use of different antihypertensive medication strategies has no influence on the outcome of patients with nonsurgical TBAD. Rather, adequate control of BP is the critical factor and more than 50% of patients required more than three types of antihypertensive medication to control their BP. BP in patients with TBAD requires more long-term and closer monitoring, with a more intensive treatment. Our study suggests that the clinical practice guidelines for the management of BP in aortic dissection should be updated.

## Figures and Tables

**Table 1:** Baseline characteristics of nonoperated TBAD patients.

Variables (*n* = 106)	Number (%)
Age, year (mean ± SD)	63.4 ± 12.9
*Age group (year)*	
<65	57 (53.8)
≧65	49 (46.2)
*Sex*	
Male	87 (82.1)
Female	19 (17.9)
Current smoker	37 (34.9)
Body mass index (mean ± SD)	25.5 ± 4.7
*Body mass index group*	
Underweight	35 (33.0)
Standard	4 (3.8)
Overweight	34 (32.1)
Obesity	33 (31.1)
Follow-up time, year (mean ± SD)	2.75 ± 1.64
*Location of aortic dissection*	
Only thoracic	16 (15.1)
Thoracic and abdominal	82 (77.4)
Only abdominal	8 (7.5)
*Initial maximum aortic diameter, cm (mean ± SD)*	4.06 ± 0.82
Thoracic aorta (*n* = 98)	4.11 ± 0.75
Abdominal aorta (*n* = 90)	3.23 ± 0.72
*Initial maximum aortic diameter group*	
<30 mm	8 (7.6)
30–39 mm	47 (44.3)
40–49 mm	38 (35.9)
≧50 mm	13 (12.3)
*Baseline blood pressure (mean ± SD)*	
SBP	129.2 ± 14.5
DBP	76.3 ± 9.7
MAP	93.9 ± 10.4
*Baseline heart rate (mean ± SD)*	
Heart rate	71.4 ± 9.3
*Comorbidities (before index day 1 year)*	
Hyperlipidemia	20 (18.9)
Hypertension	97 (91.5)
Diabetes mellitus	19 (17.9)
Chronic obstructive pulmonary disease	13 (12.3)
Heart failure	7 (6.6)
Coronary artery disease	13 (12.3)
Cerebrovascular disease	16 (15.1)
Chronic kidney disease	14 (13.2)
Cancer	10 (9.4)

**Table 2:** Blood pressure and heart rate control situations.

Follow-up (year)	1	2	3	4	*p* for trend
	*n* = 106	*n* = 77	*n* = 55	*n* = 33	
*Blood pressure control, mean ± SD*					
Poor BP control, *n* (%)^a^	21 (19.8)	20 (26.0)	19 (34.6)	16 (48.5)	<0.001
SBP	148.8 ± 9.1	149.7 ± 10.4	147.2 ± 9.1	150.5 ± 9.1	
DBP	85.8 ± 10.9	88.0 ± 9.4	88.7 ± 6.7	90.1 ± 8.8	
Well BP control, *n* (%)	85 (80.2)	57 (74.0)	36 (65.4)	17 (51.5)	<0.001
SBP	125.2 ± 8.3	125.5 ± 10.4	124.8 ± 9.4	122.9 ± 13.6	
DBP	76.2 ± 7.1	78.2 ± 9.0	77.2 ± 7.7	74.9 ± 8.7	

*Heart rate control, mean ± SD*					
Poor HR control, *n* (%)^b^	14 (13.2)	11 (14.3)	13 (23.6)	8 (24.2)	<0.001
Heart rate	86.5 ± 5.2	93.4 ± 11.7	86.9 ± 6.7	85.7 ± 4.3	
Well HR control, *n* (%)	92 (86.8)	66 (85.7)	42 (76.4)	25 (75.8)	<0.001
Heart rate	69.0 ± 6.2	68.4 ± 6.8	67.3 ± 7.3	69.5 ± 5.7	

SBP: systolic blood pressure; DPB: diastolic blood pressure; SD: standard deviation.

^a^Poor BP control defined as average SBP ≧ 140 mmHg or average DBP ≧ 90 mmHg.

^b^Poor heart rate control defined as heat rate ≧ 80 beat/min.

**Table 3:** Prescribing patterns of nonoperated TBAD patients.

Medication (%) *n* = 106	Before index date	After index date
*β*-*blockers*	*15 (14.2)*	*85 (80.2)*
Selective *β*_1_-blockers	9 (8.5)	71 (67.0)
Atenolol	3 (2.8)	4 (3.8)
Bisoprolol	6 (5.7)	68 (64.2)
Nonselective *β*-blockers	4 (3.8)	5 (4.7)
Propranolol	4 (3.8)	5 (4.7)
*α*- and *β*-blockers	5 (4.7)	10 (9.4)
Carvedilol	4 (3.8)	8 (7.6)
Labetalol	1 (1.0)	2 (1.9)
*ACEIs/ARBs*	*22 (20.8)*	*54 (50.9)*
ACEIs	7 (6.6)	7 (6.6)
Captopril	1 (0.9)	1 (0.9)
Fosinopril	1 (0.9)	1 (0.9)
Imidapril	0 (0)	2 (1.9)
Lisinopril	0 (0)	1 (0.9)
Perindopril	1 (0.9)	1 (0.9)
Quinapril	2 (1.9)	0 (0)
Ramipril	4 (3.8)	2 (1.9)
ARBs	18 (17.0)	49 (46.2)
Candesartan	4 (3.8)	15 (14.2)
Irbesartan	7 (6.6)	18 (17.0)
Losartan	1 (0.9)	5 (4.7)
Olmesartan	6 (5.7)	10 (9.4)
Telmisartan	1 (0.9)	1 (0.9)
Valsartan	10 (9.4)	27 (25.5)
*CCBs *	*25 (23.6)*	*94 (88.7)*
Dihydropyridine	22 (20.8)	92 (86.8)
Amlodipine	17 (16.0)	62 (58.5)
Felodipine	3 (2.8)	1 (0.9)
Lercanidipine	2 (1.9)	5 (4.7)
Nicardipine	1 (0.9)	17 (16.0)
Nondihydropyridine	5 (4.7)	7 (6.6)
Diltiazem	4 (3.8)	7 (6.6)
Verapamil	1 (0.9)	0 (0)
*Vasodilator*	*14 (13.2)*	*30 (28.3)*
*Diuretics*	*16 (15.1)*	*39 (36.8)*
*α-blockers*	*6 (5.7)*	*26 (24.5)*
*Statins*	*15 (14.2)*	*49 (46.2)*
Atorvastatin	12 (11.3)	26 (24.5)
Rosuvastatin	5 (4.7)	23 (21.7)
Fluvastatin	1 (1.0)	4 (3.8)
Pravastatin	3 (2.8)	5 (4.7)
*Other lipid-lowering agents*	*7 (6.6)*	*14 (13.2)*
*Antiplatelet agents*	*17 (16.0)*	*39 (36.8)*
Aspirin	13 (12.3)	34 (32.1)
Clopidogrel	10 (9.4)	13 (12.3)
Dipyridamole	4 (3.8)	5 (4.7)
Warfarin	2 (1.9)	2 (1.9)
Antidiabetic agents	6 (5.7)	18 (17.0)

**Table 4:** Events rate of nonoperated TBAD patients.

Time interval	Number of patients	Numbercensored	Number failed	Event rate (%)	Total person-year	Incidence per 100 person-year
*Primary endpoints (composite outcome)*						
≦6 months	18	3	15	83.3%	3.67	408.72
6 months~1 year	9	7	2	22.2%	7.60	26.32
1 year~3 years	40	30	10	25.0%	88.54	11.29
≧3 years	39	36	3	7.7%	159.06	1.89
Overall	106	76	30	28.3%	258.87	11.59

*Hospitalization associated with aortic dissection*						
≦6 months	18	4	14	77.8%	3.67	381.47
6 months~1 year	10	9	1	10.0%	7.60	13.16
1 year~3 years	43	40	3	7.0%	88.54	3.39
≧3 years	35	33	2	5.7%	159.06	1.26
Overall	106	86	20	18.9%	258.87	7.73

*All-cause mortality*						
≦6 months	6	4	2	33.3%	1.96	102.04
6 months~1 year	11	10	1	9.1%	9.19	10.88
1 year~3 years	41	34	7	17.1%	97.28	7.20
≧3 years	48	46	2	4.2%	182.94	1.09
Overall	106	94	12	11.3%	291.37	4.12

*Referred to surgery repair*						
≦6 months	10	6	4	40.0%	3.03	132.01
6 months~1 year	12	11	1	8.3%	9.05	11.05
1 year~3 years	45	43	2	4.4%	93.50	2.14
≧3 years	39	37	2	5.1%	174.00	1.15
Overall	106	97	9	8.5%	279.58	3.22

**Table 5:** Treatment strategy of antihypertensive drugs and event rate.

Treatment strategy^#^	Number of patients	%	Event rate, *n* (%)			
			Primary endpoints^＆^	Hosp.^§^	Death^*¥*^	Surgery^*£*^
*None*	11	10.4%	5 (45.5%)	3 (27.3%)	2 (18.2%)	1 (9.1%)
*Monotherapy*	10	9.4%	2 (20.0%)	1 (10.0%)	1 (10.0%)	0 (0%)
*β*-blocker	5	50.0%	0 (0%)	0 (0%)	0 (0%)	0 (0%)
CCB	1	10.0%	0 (0%)	0 (0%)	0 (0%)	0 (0%)
Others	4	40.0%	1 (25.0%)	1 (25.0%)	1 (25.0%)	0 (0%)
*Dual therapy*	31	29.3%	8 (25.8%)	6 (19.4%)	3 (9.7%)	4 (12.9%)
*β*-blocker + CCB	18	58.1%	3 (16.7%)	3 (16.7%)	0 (0%)	2 (11.1%)
*β*-blocker + Others	5	16.1%	2 (40.0%)	2 (40.0%)	2 (40.0%)	2 (40.0%)
CCB + RAS	3	9.7%	0 (0%)	0 (0%)	0 (0%)	0 (0%)
CCB + Others	4	12.9%	1 (25.0%)	1 (25.0%)	1 (25.0%)	0 (0%)
RAS + Others	1	3.2%	0 (0%)	0 (0%)	0 (0%)	0 (0%)
*Triple therapy*	42	39.6%	12 (28.6%)	9 (21.4%)	4 (9.5%)	3 (7.1%)
*β*-blocker + CCB + RAS	14	33.3%	3 (21.4%)	3 (21.4%)	0 (0%)	3 (21.4%)
*β*-blocker + CCB + Others	20	47.6%	5 (25.0%)	5 (25.0%)	1 (5.0%)	0 (0%)
*β*-blocker + RAS + Others	4	9.5%	1 (25.0%)	1 (25.0%)	2 (50.0%)	0 (0%)
CCB + RAS + Others	4	9.5%	0 (0%)	0 (0%)	1 (25.0%)	0 (0%)
*Quadruple therapy*	12	11.3%	3 (25.0%)	1 (8.3%)	2 (16.7%)	1 (8.3%)

CCB: calcium channel blockers; RAS: drugs acting on the rennin-angiotensin system.

^#^Antihypertensive drugs divided into *β*-blockers, drugs acting on the rennin-angiotensin system (including angiotensin converting enzyme inhibitors, angiotensin receptor blocker, and direct renin inhibitors), calcium channel blockers, and all other antihypertensive classes (including diuretics, *α*-blockers, vasodilators, and central *α*_2_ agonists).

^＆^All-cause mortality and admission to hospital because of aortic dissection (primary endpoint). ^§^Hospitalization associated with aortic dissection. ^*¥*^All-cause mortality. ^£^Referred to surgery repair.

**Table 6:** Univariable and multivariable analysis for primary endpoint.

Variables	Crude HR (95% CI)		*p* value	Adjusted HR^a^ (95% CI)		*p* value
*Treatment strategy*						
Monotherapy	ref			ref		
None	3.31	(0.64–17.22)	0.156	4.19	(0.54–32.31)	0.169
Dual therapy	1.12	(0.24–5.28)	0.886	1.66	(0.23–12.03)	0.614
Triple therapy	1.45	(0.33–6.51)	0.624	2.88	(0.41–20.35)	0.290
Quadruple therapy	1.07	(0.18–6.39)	0.944	2.01	(0.22–18.23)	0.535
Age (year)	1.01	(0.98–1.04)	0.475	1.00	(0.95–1.04)	0.831
*Sex*						
Female	ref			ref		
Male	0.96	(0.37–2.51)	0.937	1.43	(0.39–5.22)	0.587
Baseline maximum aortic diameter (cm)	2.11	(1.38–3.23)	<0.001^*∗*^	2.91	(1.70–4.99)	<0.001^*∗*^
Current smoker	1.13	(0.55–2.35)	0.740	0.89	(0.33–2.39)	0.810
BMI	1.00	(0.93–1.08)	0.974	0.98	(0.89–1.08)	0.679
*Comorbidities (before index day 1 year)*						
Hyperlipidemia	1.15	(0.47–2.82)	0.763	1.33	(0.42–4.18)	0.627
Hypertension	0.95	(0.22–4.00)	0.940	0.29	(0.05–1.71)	0.169
Diabetes mellitus	1.06	(0.40–2.80)	0.901	1.05	(0.32–3.44)	0.931
COPD	1.63	(0.67–4.00)	0.283	1.03	(0.26–4.02)	0.966
Heart failure	2.04	(0.71–5.85)	0.187	0.97	(0.22–4.28)	0.964
Coronary artery disease	1.68	(0.69–4.10)	0.258	2.06	(0.49–8.70)	0.326
Cerebrovascular disease	0.97	(0.34–2.78)	0.952	0.46	(0.13–1.66)	0.237
Chronic kidney disease	1.90	(0.77–4.65)	0.161	2.16	(0.68–6.84)	0.193
Cancer	1.55	(0.47–5.17)	0.472	2.10	(0.48–9.25)	0.326
*Poor blood pressure and heart rate control*						
Poor blood pressure control	0.94	(0.36–2.46)	0.892	0.63	(0.20–1.99)	0.431
Poor heart rate control	0.99	(0.35–2.85)	0.990	0.68	(0.16–2.88)	0.600

HR: hazard ratio; CI: confidence interval; BMI: body mass index; COPD: chronic obstructive pulmonary disease.

^a^Adjusted for treatment strategy, age, sex, initial maximum aortic diameter, current smoker, BMI, all of comorbidity, poor blood pressure, and poor heart rate control.

^*∗*^ *p* < 0.05.
